# Research about the optimal strategies for prevention and control of varicella outbreak in a school in a central city of China: based on an SEIR dynamic model

**DOI:** 10.1017/S0950268819002188

**Published:** 2020-03-17

**Authors:** Wen-ting Zha, Feng-rui Pang, Nan Zhou, Bin Wu, Ying Liu, Yan-bing Du, Xiu-qin Hong, Yuan Lv

**Affiliations:** Key Laboratory of Molecular Epidemiology of Hunan Province, School of Medicine, Hunan Normal University, Changsha, Hunan 410081, People's Republic of China

**Keywords:** Isolation, SEIR model, vaccine, ventilation and disinfection, varicella

## Abstract

Varicella is an acute respiratory infectious diseases, with high transmissibility and quick dissemination. In this study, an SEIR (susceptible-exposed-infected-recovered) dynamic model was established to explore the optimal prevention and control measures according to the epidemiological characteristics about varicella outbreak in a school in a central city of China. Berkeley Madonna 8.3.18 and Microsoft Office Excel 2010 software were employed for the model simulation and data management, respectively. The result showed that the simulated result of SEIR model agreed well with the reported data when *β* (infected rate) equal to 0.067. Models showed that the cumulative number of cases was only 13 when isolation adopted when the infected individuals were identified (assuming isolation rate was up to 100%); the cumulative number of cases was only two and the TAR (total attack rate) was 0.56% when the vaccination coefficient reached 50%. The cumulative number of cases did not change significantly with the change of efficiency of ventilation and disinfection, but the peak time was delayed; when *δ* (vaccination coefficient) = 0.1, *m* (ventilation efficiency) = 0.7 or *δ* = 0.2, *m* = 0.5 or *δ* = 0.3, *m* = 0.1 or *δ* = 0.4 and above, the cumulative number of cases would reduce to one case and TAR would reduce to 0.28% with combined interventions. Varicella outbreak in school could be controlled through strict isolation or vaccination singly; combined interventions have been adopted when the vaccination coefficient was low.

## Introduction

Varicella, commonly caused by varicella-zoster virus (VZV), is an acute respiratory infectious disease characterised by pruritic macules, papules and blisters, often accompanied by headache, fever, sore throat or other respiratory infection symptoms [1]. Varicella outbreaks often occur in schools in children with high transmissibility and quick dissemination [2]. In the USA, 90% of children under 15 years old have been exposed to VZV, and at least 90% of children over 9 years old in Germany or Belgium showed positive varicella serum antibodies [3]. In China, about 77.06% of varicella outbreaks occurred in primary schools [4], which could significantly affect the health of students, the provision of education in schools and the normal life of families [5]. The prevention and control of varicella outbreak have become the focus of health work in schools [6].

Currently, there is no specific treatment for varicella, the main prevention and control strategies of varicella outbreaks included vaccination, closure and isolation, window ventilation, wiping or spraying disinfection [7]. But due to ethical issues, limited research has been conducted regarding the comparative effectiveness of control measures for varicella [8], and it is difficult to evaluate the effectiveness of preventive and control measures through traditional epidemiological methods. Dynamic model, such as the susceptible-infected-recovered (SIR) model which successfully simulates the prevalence of infectious diseases [9, 10], has been used to evaluate the efficacy of preventive and control measures for infectious diseases. But no SIR model of varicella in central China has been researched. Thus, in this study, a susceptible-exposed-infected-recovered (SEIR) epidemic dynamics model was established to explore the optimal prevention and control measures according to the epidemiological characteristics about varicella outbreak in the school in ChangSha, a central city of China, which could provide a reference for dealing with similar incidents in other areas and formulate the prevention and control strategies of varicella outbreak in school.

## Methods

### Data collection

Data on varicella outbreak came from a public primary school in a central city in China reported by the Center for Disease Control and Prevention (CDC) of Changsha in the year 2016. The school contained six grades, eight classes, a total of 360 people. Students attend classes 5 days a week (from Monday to Friday), and boiled water was unified supplied by the school. Varicella cases were diagnosed clinically based on the history of infectious agent exposure and clinical features, such as pruritic macules, papules, blisters and respiratory infection symptoms.

### Models

#### Model of varicella outbreak without interventions

**Fig. 1.**
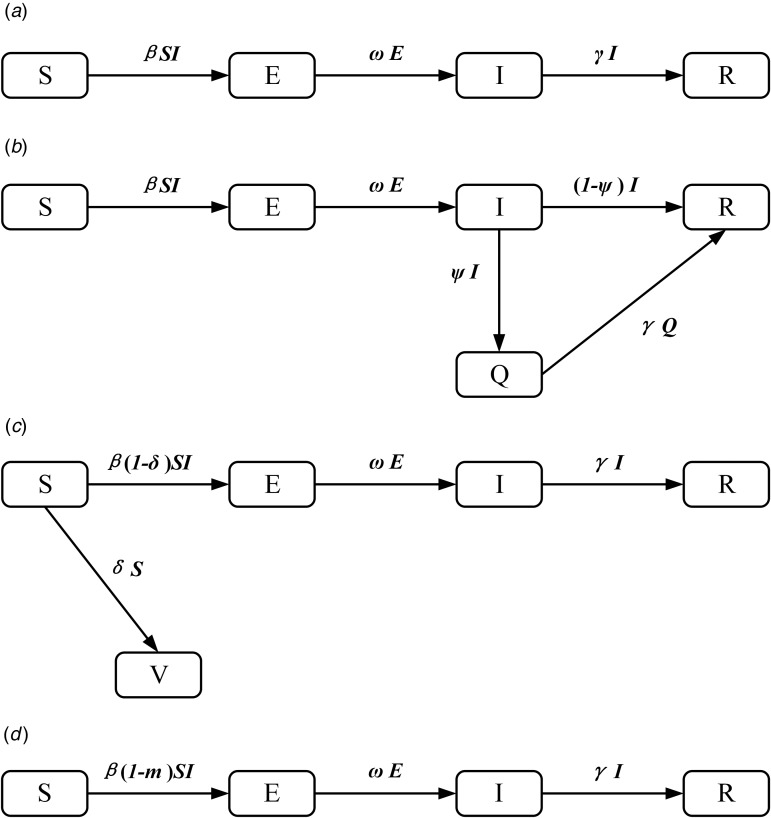
Flow chart of models of varicella outbreak in school.

An SEIR model [11, 12] was established to simulate the transmission of varicella in school without any intervention. Population in this model was divided into four categories according to the disease status (Fig. 1a): susceptible (S), exposed (E), infected (I) and recovered (R). The model was developed based on the following facts or assumptions, which assumed that some individuals moved among categories because of infection or recovery: (1) The population was defined as closed and stable, because the varicella outbreak usually occurred in school in which birth, death and migration were negligible; (2) susceptible person was assumed to have an equal infected rate (*β*) with the disease; (3) after infection, the exposed person (E) would turn to infected individuals (I) after a certain exposed period (1/*ω*), the number of newly infected individuals per unit time was *ω*E; (4) *γ* represented the recovery rate, and 1/*γ* meant the infective period, the number of newly recovered individuals per unit time was *γ*I; (5) the fatality rate of both varicella and asymptomatic infection was ignored. The corresponding model equations are as follows, *dS*/*dt*, *dE*/*dt*, *dI*/*dt* and *dR*/*dt* denoted the number of individuals (*n*) at time *t* in the corresponding categories:1
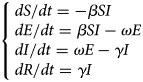

#### Model of varicella outbreak with case isolation

Isolation would be adopted when the infected individuals (I) were identified. We assumed that the isolation rate was *ψ*, *ψ*I individuals have been isolated and turned to quarantine (Q), which were not infectious. Population in the SEIQR model was divided into five categories (Fig. 1b), and the corresponding model equations are as follows:2
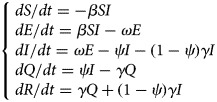


#### Model of varicella outbreak with vaccination

The SEIRV model was constructed by vaccinating susceptible people, who were separated from susceptible people and turned to vaccination (V) directly because of their immunity to varicella. Assuming that the vaccination coefficient was *δ*, the number of susceptible (S) individuals turned to exposed (E) at *t* time were (1–*δ*)S*β*I. Population in this model was divided into five categories (Fig. 1c), and the corresponding model equations are as follows, the epidemic trend under different vaccination coefficients would be simulated by adjusting *δ*.3
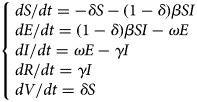


#### Model of varicella outbreak with ventilation and disinfection

Ventilation and disinfection (spraying or wiping the classroom, corridor floor and public facilities with ‘84’ disinfectants or disinfecting the air through oxyacetic acid and ultraviolet lamp) were adopted to reduce the infected rate (*β*) of varicella virus [13, 14]. Assuming that *m* was the efficiency of ventilation and disinfection to eliminate the varicella virus, the number of individuals in some categories in this model would change. The model is shown in Fig. 1d and the corresponding equations are as follows:4
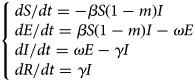


#### Modelling combinations of the intervention strategies

For the varicella outbreak in a school, we simulated the combinations of intervention strategies (isolation, vaccine, ventilation and disinfection) to explore the influence on the control of varicella outbreak in school.5
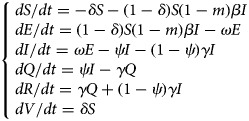


### Parameter estimation

There were six parameters in all models in this study, which were infected rate (*β*), latency coefficient (*ω*), removal rate (*γ*), isolation rate (*ψ*), vaccination coefficient (*δ*), disinfection and ventilation efficiency (*m*). The parameters *ω*, *γ*, *ψ* and *δ* could be obtained by combining the actual situation with literatures, while *β* could be obtained by fitting the actual data of model (1) and typical outbreak cases. Literature analysis showed that the exposure period of varicella is 10–21 days [15]; in this study, we assumed the exposure period (1/*ω*) as 21 days, so *ω* would equal to 1/21. Usually, the infected period of varicella was from 1 to 2 days before the eruption to scab formation; however, students would take medical measures once they have fever, rash or other symptoms; so, the infected period of varicella in school was usually shorter than the natural infection period. In this study, we assumed the isolation rate *ψ* was equal to 100%, the infection period (1/*γ*) was 3 days and the recovery rate *γ* was equal to 1/3 [16 17]. Combining with the actual situation, in order to explore the influence of vaccination coefficient (*δ*) and the efficiency of ventilation and disinfection (*m*) on the control of epidemic situation of varicella outbreak in school, we have set *δ* as 0.1, 0.2, 0.3, 0.4, 0.5, 0.6, 0.7 and *m* as 0.1, 0.3, 0.5, 0.7, 0.9, respectively. All parameters and initial values of each category are listed in Table 1.

**Table 1.** List of parameters and initial values of each category in models

Parameters and initial values	Description	Unit	Range	Value	Method
*β*	The infection rate	100%	0–100%	0.067	Curve fitting
*ω*	Latency coefficient (reciprocal of exposure period)	Day^−1^	1/10–21	0.047(1/21)	Epidemiological characteristics and actual situation
*γ*	Removal rate (reciprocal of infection period)	Day^−1^	1/2–15	0.340(1/3)	Epidemiological characteristics and actual situation
Ψ	The isolation rate	100%	0–100%	100%	Actual situation
*δ*	The vaccination coefficient	100%	0–100%	01–0.7	References and actual situation
m	Disinfection and ventilation efficiency	100%	0–100%	0.1,0.3,0.5,0.7,0.9	References and actual situation
S_0_	Number of susceptible individuals (S) at *t* = 0	−	0-	359	Actual situation
E_0_	Number of exposed individuals (E) at *t* = 0	−	0-	0	Actual situation
I_0_	Number of infected individuals (I) at *t* = 0	−	0-	1	Actual situation
R_0_	Number of recovered individuals (R) at t = 0	−	0-	0	Actual situation
Q_0_	Number of quarantine (isolation) individuals (Q) at *t* = 0	−	0-	0	Actual situation
V_0_	Number of vaccinational individuals (V) at *t* = 0	−	0-	0	Actual situation

### Simulation methods

We fitted the data from the varicella outbreak in a school in a central city of China to an SEIR model curve to estimate *β* and then simulated the effects of isolation, vaccine, ventilation and disinfection in the outbreak. Berkeley Madonna 8.3.18 and Microsoft Office Excel 2010 software were employed for the model simulation and data management, respectively. Graphpad prism 5 was used for the figure development, while the curve fitting problem was solved by the Runge–Kutta fourth-order method, with a tolerance of 0.001. A goodness-of-fit test (*χ*2 test) was performed using the IBM-SPSS software, in which the significance level was *α* = 0.05.

### Intervention assessment

The total attack rate (TAR), cumulative number of cases and duration of outbreak (DO, duration in days from index case to last case) were used to assess the effectiveness of control interventions in the varicella outbreak. Epidemic curves were used to evaluate the effectiveness using the strategies that are compared with the actual situation.

## Results

### Epidemiological features of the outbreak

**Fig. 2.**
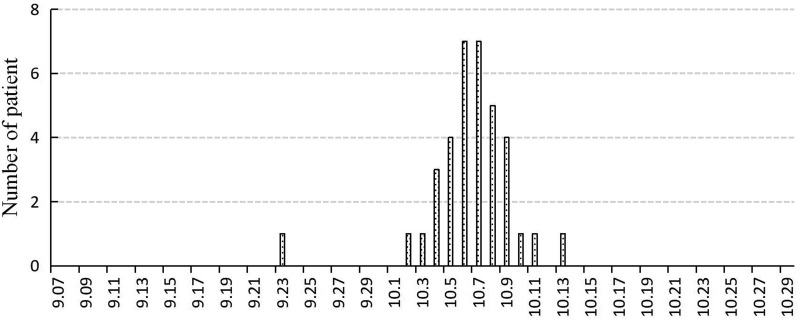
The distribution of time among students in the outbreak.

From September 23 to October 29 in the year 2016, 35 students in a school of Changsha had been diagnosed with varicella, the incidence rate was 9.72%. The first case was a fourth-grade girl, who developed symptoms on September 23. Thirty-five patients (16 boys and 19 girls) ranged from 5 to 11 years old and most of them were in the fourth grade (44.4%) and fifth grade (25.0%). Most cases were concentrated from September 23 to October 11, and no new cases of varicella were reported in the school and surrounding areas, all of them were cured as of 27 October 2016. (During this period, the school did not adopt targeted prevention and control measures.) The distribution of time among students of varicella outbreak was shown in Figure 2.

### Results of curve fitting

**Fig. 3.**
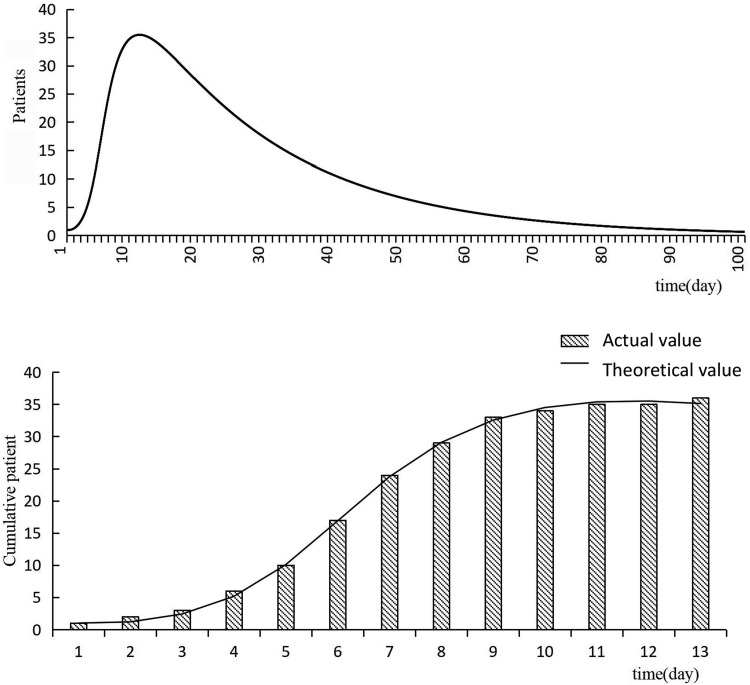
The result of curve fitting of outbreak data and SEIR model.

We set the simulation period of this study to 100 days, and the simulation result showed that the number of cases increased heading into the peak in the first 13 days, then decreased until no cases existed, indicating that the outbreak was complete (Fig. 3). The results of curve fitting of the outbreak data and SEIR model showed that the simulated result agreed well with the reported data when *β* is equal to 0.067 (*R*^2^ = 0.816, *P* < 0.01), there was no significant difference between the theoretical value and the actual value (*χ*^2^ = 0.608, *P* > 0.05).

### Case isolation

**Fig. 4.**
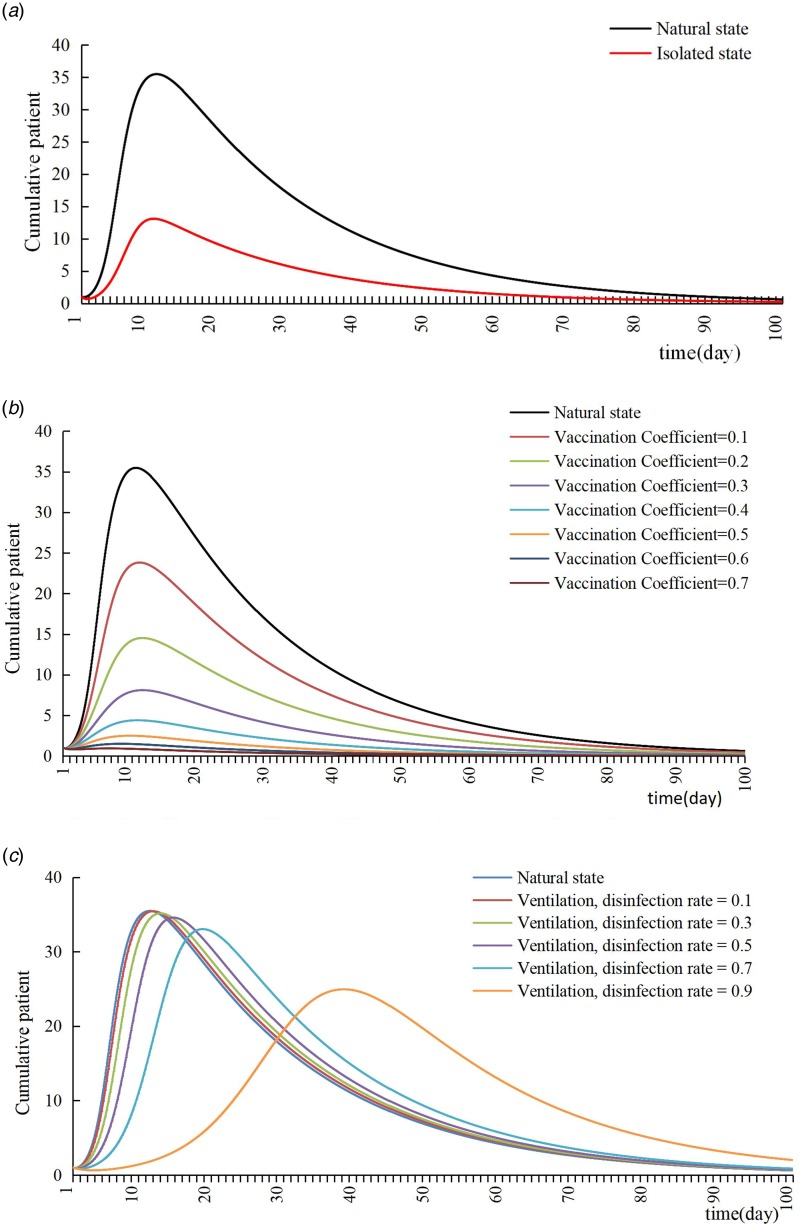
The control effect of isolation, vaccination, ventilation and disinfection.

SEIQR model showed that the cumulative number of cases would drop to 13 when isolation started from the date of discovery of patients (assuming isolation rate was up to 100%). The cumulative number of cases was significantly less than the actual situation, which meant that strict isolation of patients could effectively reduce the number of cases (Fig. 4a).

### Vaccination

SEIRV model showed that the cumulative number of cases would drop to two and the TAR would drop to 0.56% when the vaccination coefficient reached 50%, which meant that the epidemic situation could be well controlled through vaccination, and the higher the vaccination coefficient, the better the epidemic control effect (Fig. 4b).

### Ventilation and disinfection

SEIR model showed that when the effective rates of ventilation and disinfection were 10%, 30%, 50%, 70% and 90%, the cumulative number of cases were 35, 35, 34, 33 and 25, and the duration of the epidemic was 19, 20, 23, 26 and 46 days, respectively. With the change of ventilation and disinfection efficiency, the cumulative number of patients did not change significantly, but the peak time was delayed. Only when the disinfection efficiency reached 90%, the cumulative number of patients could be slightly reduced, but the duration of the epidemic was also significantly prolonged; ventilation and disinfection had no obvious effect on the control of varicella outbreak in school (Fig. 4c).

### Combined intervention

**Fig. 5.**
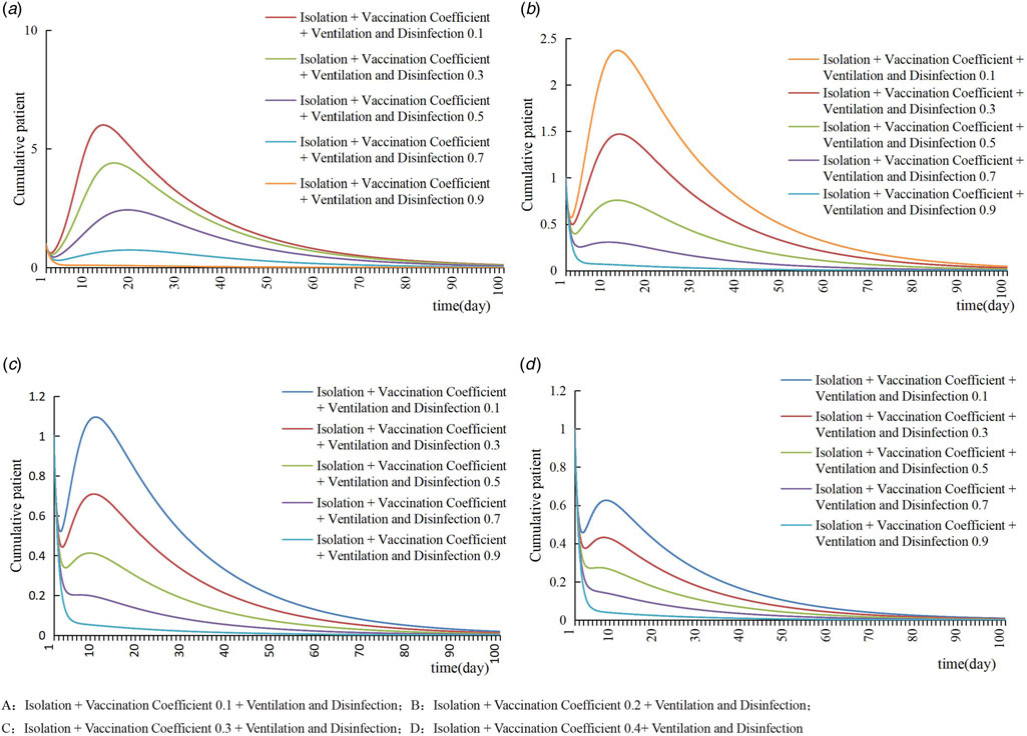
The control effect of combined intervention.

The cumulative number of patients and TAR could be significantly reduced with combined interventions. When *δ* = 0.1, *m* = 0.7 or *δ* = 0.2, *m* = 0.5 or *δ* = 0.3, *m* = 0.1 or *δ* = 0.4 and above, the cumulative number of cases would reduce to one case and TAR would reduce to 0.28%, the varicella outbreak could be controlled well (Table 2 and Fig. 5).

**Table 2.** The effect of combined intervention on the control of varicella outbreak

Interventions	DO (days)	Cumulative number of cases	TAR (%)
Actual status (without intervention)	20	35	9.72
Isolation + vaccination(*δ* = 0.1) + ventilation and disinfection			
*m* = 0.1	21	6	1.67
*m* = 0.3	23	4	1.11
*m* = 0.5	23	4	1.11
*m* = 0.7	24	1	0.28
*m* = 0.9	11	1	0.28
Isolation + vaccination(*δ* = 0.2) + ventilation and disinfection			
*m* = 0.1	19	2	0.56
*m* = 0.3	20	2	0.56
*m* = 0.5	20	1	0.28
*m* = 0.7	11	1	0.28
*m* = 0.9	10	1	0.28
Isolation + vaccination(*δ* = 0.3) + ventilation and disinfection			
*m* = 0.1	17	1	0.28
m = 0.3	17	1	0.28
*m* = 0.5	17	1	0.28
*m* = 0.7	11	1	0.28
*m* = 0.9	11	1	0.28
Isolation + vaccination(*δ* = 0.4) + ventilation and disinfection			
*m* = 0.1	16	1	0.28
*m* = 0.3	16	1	0.28
*m* = 0.5	15	1	0.28
*m* = 0.7	12	1	0.28
*m* = 0.9	10	1	0.28

## Discussion

Varicella is very contagious and often occurs in children, which has a great negative effect on school [18]. In this study, an SEIR epidemic dynamics model was established to explore the optimal prevention and control measures according to the epidemiological characteristics of varicella for controlling future outbreaks, which is the first time to establish an SEIR model of varicella outbreak in the school of China.

There were three common ways to prevent and control varicella, which were vaccination, isolation, ventilation and disinfection. Models showed that the epidemic situation of varicella outbreak could be well controlled through isolation or vaccination singly, but ventilation and disinfection alone had no obvious effect on the control of varicella outbreak in school. Strict isolation could control the outbreak of varicella, but due to the influence of diagnosis conditions and other factors, the cases with varicella we have found in the first time only accounted for a part of all patients, and the isolation rate was difficult to reach 100% [19]. Therefore, in order to control the development of varicella, it is necessary to take initiative of students and parents to report the disease timely or monitor the temperature of students every day during the period of high incidence of varicella to raise the isolation rate in the actual situation. The varicella vaccine was approved and marketed in the USA in 1995, but the vaccination coefficient in China was not high because the varicella vaccine was voluntary. It took 15 days to produce antibodies after vaccination [20 21]; therefore, it is necessary to improve the vaccination coefficient of varicella for students, a high-risk group, and they should be vaccinated early at least 15 days before the period of high incidence of varicella, which usually is in the 16^th^ and 45^th^ weeks per year [22]. In the actual prevention and control work, ventilation and disinfection are also one of the important prevention and control measures. However, the results of this study indicated that the epidemic situation has not been effectively alleviated by reducing the density of VZV in the environment through ventilation and disinfection measures alone, which may be due to the fact that varicella virus mainly comes from the herpes fluid in the skin lesion of the patient's eruption site or the respiratory secretion of the patient [4].

Models showed that the cumulative number of patients and TAR could be significantly reduced with combined interventions. On the basis of strict case isolation, the vaccination coefficient of varicella vaccine among students was investigated. In the actual work, when the vaccination coefficient was lower than 0.4, combined interventions (isolation plus vaccination plus ventilation and disinfection) could be taken to intervene comprehensively.

We modelled a theoretical isolation and did not consider all actual interventions; therefore, differences remained between the simulation and the actual outbreak, which is the limitation in our study.

## Conclusions

Varicella outbreak in the school of China could be well controlled through strict isolation or vaccination singly, but ventilation and disinfection alone had no obvious effect on the control of varicella outbreak in school; combined interventions of isolation, vaccination, ventilation and disinfection could be adopted when the vaccination coefficient was low.
